# Loneliness, Emotional Fatigue, Social Dysfunction, and Suicide Risk in Ecuadorian University Students: A Mediation Analysis

**DOI:** 10.11621/pir.2026.0205

**Published:** 2026-06-30

**Authors:** Rodrigo Moreta-Herrera, Paulina Gordón-Villalba, Jose A. Rodas, Evelyn Cuesta-Andaluz, Priscila Revelo-Sánchez

**Affiliations:** a Pontifical Catholic University of Ecuador, Ambato, Ecuador; b Technical University of Ambato, Ecuador; c Espíritu Santo University, Ecuador; d University College Dublin, Ireland; e Technological University Indoamérica, Ambato, Ecuador; f Armed Forces University – ESPE, Ecuador

**Keywords:** emotional fatigue, social dysfunction, suicide risk, mediation, loneliness

## Abstract

**Background.:**

Suicidal risk in university students does not require analysis solely from clinical parameters; it is also necessary to understand it from the perspective of psychosocial factors and the everyday life situations to which the students are mostly exposed. Furthermore, it is a phenomenon with multiple causes, so studying it by integrating the complexity of various actors can help us understand not only direct dynamics but also indirect, multifaceted ones.

**Objective.:**

To estimate a sequential and latent multiple mediation model of emotional fatigue and social dysfunction in the relationship between loneliness and suicidal risk in Ecuadorian university students.

**Design.:**

A descriptive, cross-sectional, multiple mediation study using structural equation modelling (SEM). The sample comprised 943 undergraduate students (64.4% women, 35.6% men) from six universities (61.1% public) in four Ecuadorian cities, aged between 18 and 47 years (*M* = 21.62, *SD* = 3.81).

**Results.:**

Loneliness, emotional fatigue, and social dysfunction are latent predictors of suicidal risk, explaining 60% of its variance. Loneliness exerts both a direct effect on suicide risk and an indirect effect through the sequential multiple mediation of emotional fatigue and social dysfunction.

**Conclusion.:**

Academic risk factors such as loneliness, emotional fatigue, and social dysfunction are key attributes to consider for preventing suicidal behavior in university students. These predictors can dynamically interact to generate both direct and indirect effects, allowing for a better understanding of the complexity of this phenomenon.

## Introduction

Suicide is a major global public health problem. Over 700,000 people die each year from this condition, and among young people, it is the fourth leading cause of death (World Health Organization, 2021). In Ecuador, an estimated 1,000 individuals commit suicide annually, and for every completed suicide, there are at least 20 attempts (Ministerio de Salud Pública del Ecuador [MSP], 2023). Therefore, it is necessary to study this phenomenon from its early stages to understand its underlying mechanisms, assess the existing risk, and prevent the act itself in a timely manner. Suicide risk is a condition that affects various populations to varying degrees, although university students constitute a notably vulnerable group. The loss of hope and life satisfaction are already indicators of impaired academic satisfaction (Santillán-García et al., 2025). Furthermore, academic, social, and personal demands can make students susceptible to mental health problems (Castillo-Gonzáles et al., 2026; Moreno-Montero et al., 2023), including suicide risk (Lapo-Talledo et al., 2025), with reported rates of 9.7% for suicidal ideation and .7% for suicide attempts (Crispim et al., 2021; Oleas et al., 2025). Thus, this condition does not depend on a single factor, but on a convergence of components related to emotional distress, relational difficulties, and psychosocial vulnerability. While factors associated with mental health disorders—such as stress, anxiety, depression, sleep disturbances, and general emotional distress—have been identified (Gutiérrez et al., 2021; Ward et al., 2022), contextual and daily aspects, like financial pressure and economic difficulties, also demonstrate a significant connection (Sheldon et al., 2021). Moreover, several of these elements are part of daily academic life and can be determining factors even without presenting as clinical symptoms.

### Psychosocial Factors Associated with Suicide Risk in University Students

Although factors such as life satisfaction (Moreta-Herrera et al., 2025), well-being, social support (Lascano-Arias et al., 2025), and personal strengths (Bahamón et al., 2023) are considered protective against suicidal behavior, certain factors in academic life require attention, as their unmitigated manifestation could increase suicide risk. Perceived loneliness, academic fatigue, and functional problems warrant study due to their established connections and require more in-depth analysis.

Firstly, loneliness is understood as a subjective experience of social relationships failing to meet the need for interpersonal connection or closeness (Hawkley & Cacioppo, 2010). Unlike isolation or abandonment “being alone”, loneliness involves the distress of “feeling alone” even when maintaining social relationships or support networks. This experience is prevalent among university students, often reported at high intensities (Conti et al., 2023), and is generally associated with a lack of university identity (Zhou et al., 2023). Regarding suicide-related issues, moderate positive correlations with suicide risk are reported (Jin et al., 2025), particularly with suicidal ideation (Moreta-Herrera et al., 2026; Oh et al., 2025), and it is even considered a positive predictor (Hamdan-Mansour et al., 2024).

Alongside loneliness is academic emotional fatigue, which refers to the mental exhaustion stemming from prolonged study demands, performance pressure, and academic overload (Schaufeli et al., 2002)—that is, feeling overwhelmed by academic tasks (Campos et al., 2005; Wu et al., 2023). Role ambiguity, conflicts, high pressure, and competitiveness are factors associated with this fatigue (Labrague & Ballad, 2021), which is often pronounced in university students. Its intensity can vary depending on contextual factors (Moreta-Herrera et al., 2022), negatively affecting mental health and potentially leading to self-harm and suicidal behaviors (Lin et al., 2021). In this regard, evidence shows significant positive relationships with suicide risk (Aquino-Canchari et al., 2025; Kleiman et al., 2018), and it appears to act as a positive predictor (Noyosase Igbineweka et al., 2025).

Finally, social dysfunction refers to difficulties in adequately performing everyday roles and normal daily activities. It is conceived as an indicator of the multidimensional perception of psychological distress (Goldberg, 1978). While its presence among university students is usually low (Affan et al., 2025; Yus et al., 2025), this element could be key for adequate academic performance, although available evidence is rather limited. Regarding its relationship with suicide risk, specific information on this construct is lacking; however, multidimensional studies of psychological distress show mild positive correlations (Nowakowska-Domagała et al., 2023), without estimating predictive evidence.

Summarizing the findings, loneliness, emotional fatigue, and social dysfunction are psychosocial factors present in the daily dynamics of the university population that impact psychological sensitivity. The evidence of how these interact with suicide risk provides a clear warning for decision-making, particularly concerning the reinforcement of prevention strategies and mental health support to minimize the overall risk of suicidal behavior.

### The Present Study

While loneliness, emotional fatigue, and social dysfunction are variables connected to suicide risk among university students, most studies have examined them in isolation (focusing primarily on correlations). This provides a narrow and unrealistic view of their interaction. Thus, it is necessary to explore the interconnected triggering factors that operate jointly to provide a comprehensive view of suicide risk. Studies that yield explanatory models of these multiple connections would offer further insight into the mechanisms of risk through everyday, non-clinical factors present in students’ daily lives.

Proposing explanatory models helps to elucidate the mechanisms of action of various predictor variables on a criterion variable, particularly by estimating direct and indirect effects through the inclusion of mediating variables (Gunzler et al., 2013; Lange et al., 2021). Among the variables of interest, loneliness is the most prominent, showing the strongest evidence of relevant covariance and predictive ability for suicide risk in university students. Furthermore, it shares a sustained correlation with emotional fatigue (Kadanji et al., 2025; Shim & Go, 2025) and social dysfunction (Tsiridis et al., 2023). This suggests that loneliness likely exerts not only a direct effect on suicide risk, as previously established (Hamdan-Mansour et al., 2024), but also independent indirect effects via emotional fatigue and social dysfunction. There may even be an unexplored sequential indirect effect, given the positive correlation between emotional fatigue and social dysfunction (Theofilou et al., 2022).

**Figure 1.**
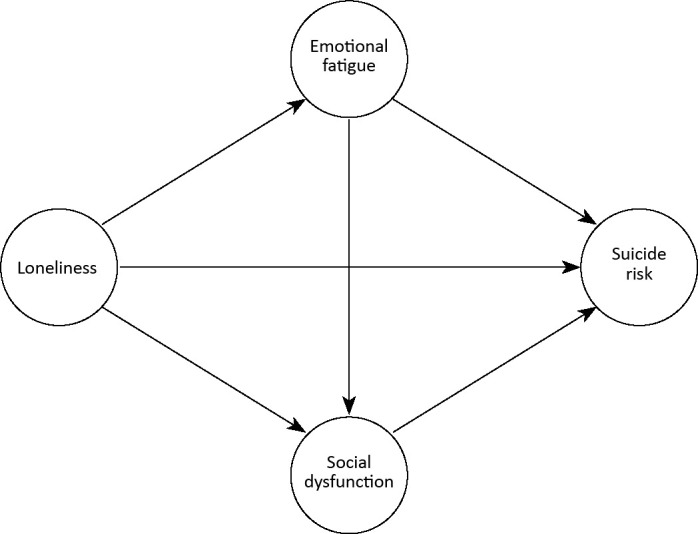
Explanatory predictor and multiple mediation model

Consequently, developing an explanatory model of suicide risk that identifies loneliness, emotional fatigue, and social dysfunction as multiple predictors—while simultaneously assessing both direct and indirect effects (see **Figure 1**)—would enrich existing knowledge of suicidal behavior based on daily psychosocial factors. This approach allows for a more comprehensive examination of suicide risk without requiring the presence of significant clinical indicators of mental distress, such as symptoms of anxiety, depression, or stress. University students generally face particular, everyday stressors that do not always initially manifest as mental health disorders; thus, by examining these factors from a transdiagnostic perspective, mechanisms related to mental health deterioration and even suicide risk can be identified. This can help establish a framework for interpreting suicide risk from a non-pathological or non-clinical perspective, contributing ideas, input, and resources to preventive intervention processes or to the identification of risk in subclinical stages.

Therefore, based on these premises, the following research objectives are proposed: a) to examine the latent relationships between loneliness, academic emotional fatigue, social dysfunction, and suicide risk in a sample of university students; b) to determine the predictive effect of loneliness, emotional fatigue, and social dysfunction on suicide risk through a structural multiple predictor model; and c) to evaluate the latent mediating role of academic emotional fatigue and social dysfunction in the relationship between loneliness and suicide risk. Based on these objectives, it is hypothesized that loneliness will be positively associated with academic emotional fatigue, social dysfunction, and suicide risk (H_1_); that loneliness, academic emotional fatigue, and social dysfunction will jointly predict suicide risk (H_2_); and that academic emotional fatigue and social dysfunction will mediate the relationship between loneliness and suicide risk (H_3_).

## Method

### Design

The study employed a descriptive, predictive, multiple mediation, and cross-sectional design (Ato et al., 2013) using structural equation modelling (SEM) techniques in a sample of Ecuadorian university students.

The study included 943 participants (64.4% women, 35.6% men) aged 18 to 47 years (*M* = 21.62, *SD* = 3.81); 89.1% are between 18 and 25 years old (typical ages for university studies), and 10.1% are over 25 years old (less common ages for undergraduate university studies). Ethnically, 89.3% identified as mestizo, while the remaining 10.7% identified as indigenous, rural residents, or Afro-Ecuadorian. Additionally, 73.3% resided in urban areas and 26.4% in rural areas. The undergraduate students came from six universities (61.1% public) in four Ecuadorian cities. Furthermore, 37.2% combined their studies with work, and 23% reported being at risk of poor academic performance. A non-probabilistic convenience sampling method was used based on the following inclusion criteria: being of legal age and providing informed consent, voluntary participation, and being officially enrolled at the participating universities.

### Measures

*University of California Los Angeles Loneliness Scale* (UCLA-LS; Russell et al., 1978) in its 10-item Spanish version (Velarde-Mayol et al., 2016). This measure assesses subjective perceived loneliness. It consists of 10 items rated on a 5-point Likert scale ranging from 0 (*never*) to 4 (*always*). Although it lacks normative interpretative values, higher scores indicate a greater perception of loneliness. Regarding its psychometric properties, the Spanish version of the UCLA-LS (Velarde-Mayol et al., 2016) has a unidimensional structure and demonstrated high internal consistency, *α* = .95.

*Emotional Exhaustion Scale* (ECE, for its acronym in Spanish; Campos et al., 2005), adapted for Ecuadorian university students (Moreta-Herrera et al., 2022). This scale evaluates effective academic emotional fatigue arising from academic demands.

It comprises 10 items rated on a 5-point Likert scale from 0 (*never*) to 4 (*always*). Higher scores denote greater emotional fatigue. The Ecuadorian version of the ECE (Moreta-Herrera et al., 2022) presents a unidimensional structure with high internal consistency, *ω* > .95.

*The Social Dysfunction dimension of the 28-item General Health Questionnaire* (GHQ-28; Goldberg, 1978), validated in Ecuadorian university students (Moreta-Herrera, Rodríguez-Lorenzana, et al., 2024), assesses the functional impact of psychological distress on an individual’s daily functioning and social environment. It includes seven items rated on a 4-point Likert scale, with higher scores reflecting greater social dysfunction. This dimension in the Ecuadorian version showed high internal consistency, *ω* = .98 (Moreta-Herrera, Rodríguez-Lorenzana, et al., 2024).

*Paykel Suicide Scale* (Paykel et al., 1974), translated into Spanish (Fonseca-Pedrero et al., 2018) and adapted for Peru (Baños-Chaparro & Ramos-Vera, 2020), analyzes the continuum of suicidal behavior, focusing primarily on suicidal ideation and planning over the past six months. It consists of five items rated on a frequency scale ranging from 0 (*never*) to 4 (*always*). Higher scores indicate a greater suicide risk. The Peruvian version (Baños-Chaparro & Ramos-Vera, 2020) used in the current study presents a unidimensional structure with correlated errors for items 4 and 5, as a portion of the variance is not explained by the latent factor. Internal consistency was acceptable, *ω* = .82.

### Procedure

Initially, institutional permissions were obtained from the participating study centers, followed by informed consent from the potential sample. Data collection was then conducted virtually using Google Forms. Prior to the assessment, participants read the consent forms detailing the study’s objectives, their roles, and relevant ethical safeguards, before voluntarily agreeing to participate. The evaluation process lasted approximately 15 minutes and took place at the participating institutions during the first semester of 2025. Upon completion, data were cleaned and systematized in electronic databases for analysis and reporting. Regarding ethical considerations, this study complied with the Declaration of Helsinki guidelines for human research. Furthermore, the overarching project adhered to institutional ethics regulations and received approval from the Institutional Review Board of the Pontificia Universidad Católica del Ecuador, Ambato campus.

### Data Analysis

Data analysis was conducted in four stages. The first involved a descriptive analysis of the variables, reporting the mean (*M*), standard deviation (*SD*), skewness (*g*_1_), and kurtosis (*g*_2_), along with the measurement range indicating the mean’s relative position against the test’s maximum and minimum scores. Univariate normality was assessed using *g*_1_ and *g*_2_ values, expecting margins of ~1.5. Multivariate normality was evaluated via Mardia’s test (1970), indicated by a lack of statistical significance (*p* > .05).

The second stage examined the factorial validity of the measures using Confirmatory Factor Analysis (CFA) based on a polychoric correlation matrix with Diagonally Weighted Least Squares (DWLS) estimation. This estimator was chosen due to the ordinal nature of the items and the lack of multivariate normality (Li, 2016). Factorial validity was verified using the Chi-square (*χ*^2^), Standardized Root Mean Square Residual (SRMR), Comparative Fit Index (CFI), Tucker-Lewis Index (TLI), and Root Mean Square Error of Approximation (RMSEA). Adequate validity was determined by a non-significant *χ*^2^(*p* > .05), CFI and TLI > .95, SRMR and RMSEA < .08, and factor loadings > .50 (Brown, 2015; Byrne, 2008; Liu et al., 2023; Moreno-Montero et al., 2023; Wolf et al., 2013). Item internal consistency was calculated using McDonald’s coefficient (ω; McDonald, 1970), with values above .70 considered adequate.

Following this, structural models were evaluated. The first involved a latent relationship model using SEM techniques to determine the intensity of covariances, expecting at least mild latent relationships (*r* > .20). The second model was a structural double mediation model, comprising loneliness as the exogenous variable (*X*), suicide risk as the endogenous variable (*Y*), and emotional fatigue (*M1)* and social dysfunction (*M*2) as mediators. This model analyzed the direct effect (*c*') of loneliness on suicide risk, the independent indirect effects of emotional fatigue (*a*1*b*1) and social dysfunction (*a*2*b*2), and the sequential effect of emotional fatigue and social dysfunction (*adb*) on this relationship. Independent and sequential mediation is supported when the indirect effects (*a*1*b*1, *a*2*b*2, and *adb*) are significant (*p* < .05). If the direct effect (*c*') ceases to be significant (*p* > .05) after including the sequential indirect effect (*adb*), the mediation is considered total; otherwise, it is considered partial (Baron & Kenny, 1986; Lange et al., 2021; Moreta-Herrera et al., 2023). Finally, the mediated proportion of the model (*adb*/*c*) was calculated to determine the percentage of the total effect accounted for by mediation. Additionally, as a regression-based model, the structural coefficient of determination (*R*^2^) was calculated to ascertain the percentage of variance in suicide risk explained by loneliness, emotional fatigue, and social dysfunction. A predictability of > .20 was expected for it to be considered moderate (Ferguson, 2016).

Statistical analyses were conducted using R software version 4.2.2 (R Core Team, 2023) within the RStudio environment, utilizing the *foreign*, *lavaan*, *MVN*, and *MBESS* packages.

## Results

### Preliminary Analysis of the Measures

*Table 1* displays the general performance of the variables of interest. Emotional fatigue is the most prominent attribute, reaching a moderate intensity among participants. This is followed by perceived loneliness at a low-to-moderate intensity, and finally, social dysfunction and suicide risk at low intensities. Since the distribution values fall within the ~1.5 range, univariate normality is assumed; however, the assumption of multivariate normality is not met due to significant results (*p* < .05) in Mardia’s test applied to skewness (g_1_) and kurtosis (g_2_).

**Table 1:** Preliminary Analysis of the Measures

Measures	M	Range	SD	g_1_	g_2_	Mardia g_1_	Mardia g_2_
UCLA	15.84	39.6%	8.50	.22	–.25	1321.24***	54.64***
ECE	21.03	52.6%	9.58	.04	–.64	1518.16***	65.53***
Social Dysfunction	14.31	26.1%	3.86	.26	–.02	747.94***	37.9***
Paykel	4.33	17.3%	4.89	1.35	1.24	2070,34***	6.39***

Notes. *** p < .001; M: mean; SD: standard deviation; g_1_: skewness; g_2_: kurtosis

### Factorial Validity of the Measures

*Table 2* presents the results of the CFA applied to each measure, a preliminary step before conducting structural analyses of latent relationships (general fit model) and predictions (structural regression and mediation analysis).

The study’s measures for loneliness (UCLA-LS), emotional fatigue (ECE), social dysfunction (with correlated errors for items 6 and 7), and suicide risk (PSS, with correlated errors for items 4 and 5) show optimal fit indices for their theoretical models. This indicates that the measures are empirically consistent with the data and, therefore, valid for use among Ecuadorian university students. Furthermore, regarding internal consistency, all obtained values exceed the reference score (ω > .70), indicating high reliability for all measures.

**Table 2:** Confirmatory Factor Analysis of the Loneliness, Emotional Fatigue, Social Dysfunction, and Suicide Risk Measures

Measures	χ_^2^_	gl	CFI	TLI	RMSEA	SRMR	ω
UCLA	312.34***	35	.995	.994	.092 [.083 – .101]	.044	.942
ECE	267.31***	35	.998	.998	.084 [.075 – .094]	.035	.960
Social Dysfunction	106.31***	13	.994	.991	.087 [.072 – .103]	.045	.799
Paykel	27.56***	4	.999	.999	.079 [.053 – .108]	.021	.929

Notes. χ^2^: chi-square; df: degrees of freedom; CFI: comparative fit index; TLI: Tucker-Lewis index; RMSEA: root mean square error of approximation; SRMR: standardized root mean square residual. *** p < .001

### Latent Relationship Analysis

**Figure 2.**
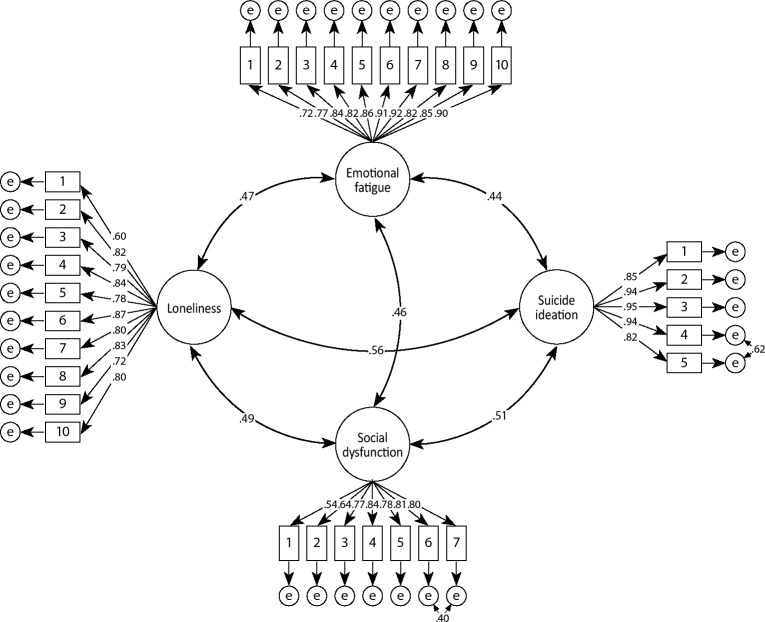
General fit model between suicide risk, loneliness, academic fatigue, and social dysfunction

*Notes. χ^2^= 1980.56; p < .001; df = 456; CFI = .995; TLI = .994; RMSEA = .060 [.057 – .062]; SRMR = .050. χ^2^*= *chi-square; df* = *degrees of freedom; CFI = comparative fit index; TLI = Tucker-Lewis index; RM-SEA = root mean square error of approximation; SRMR = standardized root mean square residual.*

**Figure 2** displays the general fit model of the variables of interest for estimating their latent relationships. All variables show positive and moderately intense covariances. The strongest association is between loneliness and suicide risk, whereas the weakest is between emotional fatigue and suicide risk.

### Predictor and Mediation Model

**Figure 3.**
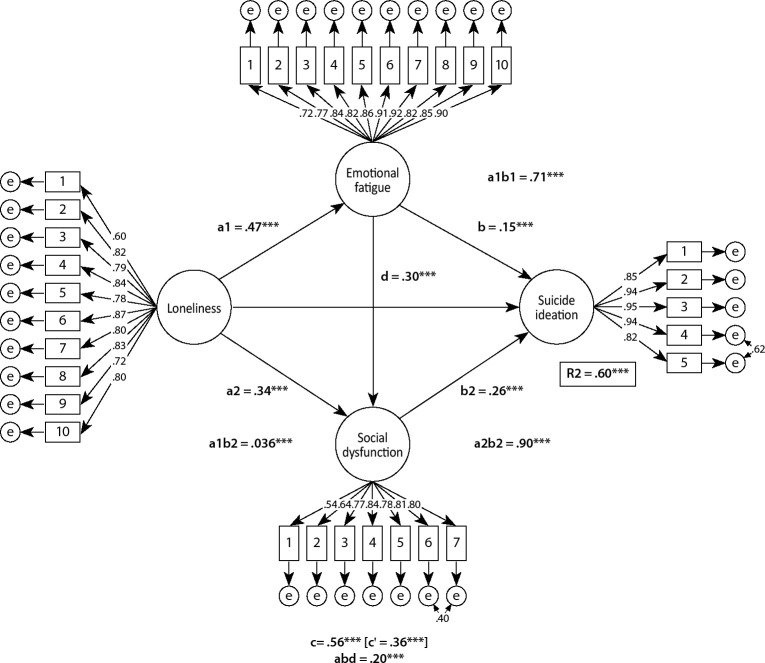
Mediation model between loneliness and suicide risk through emotional fatigue and social dysfunction

*Notes. χ^2^= 1980.56; p < .001; df= 456; CFI= .995; TLI= .994; RMSEA= .060 [.057 – .062]; SRMR= .050. χ^2^= chi-square; df = degrees of freedom; CFI = comparative fit index; TLI = Tucker-Lewis index; RM-SEA = root mean square error of approximation; SRMR = standardized root mean square residual.*

**Figure 3** illustrates the predictive model of suicide risk based on loneliness, emotional fatigue, and social dysfunction. In the latent structural regression model, loneliness, emotional fatigue, and social dysfunction explain 60% of the variance in suicide risk, representing a large effect. Thus, these three elements are considered strong positive predictors of suicide risk.

Regarding the multiple mediation analysis, loneliness exerts a substantial direct effect (*c*) on suicide risk and also presents specific indirect effects through a) emotional fatigue (*M*1); b) social dysfunction (*M*2), which has the larger effect; and c) the combination of both mediators, representing a sequential indirect effect. The total of these three indirect effects (*adb*) is .20, *p* < .001, considered moderate. Moreover, given that the direct effect (*c*') is also statistically significant (*p* < .05) after incorporating the indirect effects (*abd*), this indicates that the mediation is not complete, but only partial. Furthermore, the mediated proportion of the model indicates that 35.7% of the total effect is mediated. This implies that a portion of the relationship between loneliness and suicide risk is explained by intermediate psychological mechanisms.

Regarding the fit indices (general fit model, structural regression, and mediation), the proposed models for latent relationships and mediation yield adequate fit values. This indicates that the discrepancy error between the theoretical models and the data is relatively small, making them empirically consistent.

## Discussion

This study aimed to determine the relationship between loneliness, emotional fatigue, social dysfunction, and suicide risk in a sample of Ecuadorian university students. Furthermore, it sought to establish their joint predictive potential for suicide risk, along with the direct and indirect effects of loneliness on this condition.

Regarding the prevalence of the variables of interest, emotional fatigue was the most prominent condition among the students, demonstrating moderate intensity. This condition is widely reported among participants, aligning with previous research on university samples (Labrague & Ballad, 2021; Moreta-Herrera et al., 2022; Wu et al., 2023). It appears to be an inherent characteristic of their adaptation to adulthood, academic life associated with perceived overload, conflicts, and academic pressure, warranting further investigation. This was followed by perceived loneliness, reported at a slightly lower intensity than emotional fatigue. The data indicate that approximately 40% of cases experience some degree of loneliness or limited interpersonal closeness within their academic context. It is inferred that “feeling lonely” forms a significant part of the daily university experience at some stage. While the intensity reported in this study is lower than in some previous research showing elevated levels (Conti et al., 2023; Zhou et al., 2023), similar data in Ecuador are scarce, necessitating further research to expand upon these dynamics. Social dysfunction showed a low prevalence, indicating that only a small proportion of participants report difficulties in adequately and effectively performing everyday roles and daily activities. These results are consistent with previous studies reporting low levels (Affan et al., 2025; Yus et al., 2025). Although not characteristic of the university population, it requires monitoring as an independent construct.

Finally, suicide risk presented at a low intensity, although its prevalence remains notable (~17%) for this demographic. This indicates that the condition affects a significant proportion of students and elevates the probability of more severe suicidal behaviors (*e.g.*, suicidal planning and execution). These findings align with previous reports (Crispim et al., 2021; Oleas et al., 2025; Ward et al., 2022), highlighting an issue that demands further study and intervention mechanisms, particularly given the academic training context of this population.

Regarding the latent relationships, suicide risk correlated moderately and positively with loneliness, consistent with prior national and international research (Moreta-Herrera et al., 2026; Oh et al., 2025). Similarly, it correlated with academic emotional fatigue in the same direction and magnitude, aligning with previous findings (Aquino-Canchari et al., 2025; Kleiman et al., 2018). Lastly, it correlated moderately and positively with social dysfunction — albeit measured as a dimension of a broader construct (psychological distress)— which corresponds with previous reports (Affan et al., 2025; Yus et al., 2025). In summary, loneliness (showing the strongest relational proportion), emotional fatigue, and social dysfunction are consistently connected to suicide risk at a moderate intensity. This is particularly relevant due to their shared variance.

Unlike much of the available evidence, this study examines these three variables concurrently, providing a broader perspective on their interconnectivity. This is illustrated by the latent relationship model, which demonstrates coherence between the proposed theoretical model (see **Figure 1**) and the empirical data.

In the multiple latent prediction model, loneliness, emotional fatigue, and social dysfunction emerged as positive latent predictors of suicide risk. Together, these attributes explain 60% of the variance in suicide risk, representing a large effect (Ferguson, 2016). Given the complexity of this explanatory model, there are no prior studies for direct comparison; however, the predictability of loneliness (Hamdan-Mansour et al., 2024) and emotional fatigue (Noyosase Igbineweka et al., 2025) aligns with previous conclusions. The inclusion of social dysfunction demonstrates a novel and relevant contribution to the multifactorial understanding of suicide risk.

Delving into the specific effects, the latent multiple mediation analysis revealed that loneliness exerts a direct effect on suicide risk, alongside indirect effects via emotional fatigue and social dysfunction. A sequential effect was also observed through emotional fatigue and social dysfunction, which share covariance (Theofilou et al., 2022). These findings highlight the complex mechanisms of loneliness, suggesting that mitigating suicide risk by targeting loneliness in isolation — without addressing intermediate variables like emotional fatigue and social dysfunction — may be challenging. Furthermore, the sequential mediation indicates a probable “cascade” effect, where loneliness contributes to emotional fatigue, which in turn impairs social functioning, culminating in increased suicide risk. The significant direct effect (*c*', *p* < .05) indicates partial mediation. Therefore, both emotional fatigue and social dysfunction act as latent mediators in the relationship between loneliness and suicide risk.

Finally, the latent relationship and multiple latent mediation models demonstrated adequate fit indices (Brown, 2015; Byrne, 2008; Moreno-Montero et al., 2023; Wolf et al., 2013). This indicates coherence between the theoretical assumptions and the empirical data, allowing for generalization to reference samples of Ecuadorian university students. Notably, studies with these specific characteristics have not been conducted previously; thus, this pioneering work advances the understanding of psychosocial factors associated with suicide risk in the university population using everyday indicators of academic life. However, due to the lack of comparative literature, these findings should be interpreted with caution.

Regarding its implications, this study provides a novel explanatory model for suicide risk associated with loneliness, emotional fatigue, and social dysfunction. This yields valuable insights into understanding suicide risk through subclinical, everyday factors. Crucially, it demonstrates that intermediate psychological mechanisms explain a significant portion of the relationship between loneliness and suicide risk (*c*/*adb* = 35.7%), a finding of substantial explanatory relevance for this line of research. Consequently, this provides a practical foundation for formulating psychological intervention strategies targeting suicide risk, particularly for preventive action in subclinical and prodromal phases before severe suicidal manifestations consolidate. Interventions could therefore focus on cognitive strategies (Alvarado-Zurita et al., 2025), emotional regulation (Rodas et al., 2022), and social support based on these indicators to prevent suicide.

## Conclusions

Loneliness, emotional fatigue, and social dysfunction are positive predictors of suicide risk among university students in Ecuador. Together, these factors explain 60% of the change in the variance of suicide risk. Furthermore, considering the effects, loneliness has both a direct effect on suicide risk and an indirect effect sequentially through emotional fatigue and social dysfunction, which suggests that the relationship between loneliness and risk is explained by intervening psychological phenomena.

## Data Availability

The data associated with these results are available to those interested and can be requested from the corresponding author of the study. It is important to note that the use of the same will be used solely for research and academic purposes and under no pretext for commercial purposes.

## Limitations

The study presents several limitations that should inform future research. Firstly, while loneliness, emotional fatigue, and social dysfunction were identified as predictors of suicide risk, the cross-sectional design precludes causal conclusions. Future research should employ longitudinal predictive and mediational designs with at least three time points, or experimental methodologies. Secondly, the sample was restricted to university students, excluding other educational levels, such as secondary school students. Consequently, the findings are limited to this specific demographic. Future studies should encompass diverse educational groups, as well as academic staff and researchers. Within this same context, it is important to note that the participants have a fairly broad age range, and 10% of them are of ages less common in undergraduate university studies. This could potentially influence the prevention of suicidal behavior. Therefore, it is recommended that future studies explore strategies to control this variable or, failing that, to segment the sample. Finally, regarding the explanation of the variance in the predictive model, although an R^2^of 60% does not constitute direct evidence of variance in the common method, the use of self-report measures could increase this risk. Consequently, it is recommended to evaluate and report control procedures, such as the Harman one-factor test or analysis using marker variables. Furthermore, although the model demonstrated high explanatory power, there is insufficient evidence of multicollinearity; future research could complement the analysis by evaluating potential effects (through variance inflation factors [VIF], tolerance indices, or redundancy analysis). This would examine possible overlaps between predictors.
